# Buccodental Toxicities Induced by Tyrosine Kinase Inhibitors in Patients Diagnosed with Renal Cell Carcinoma—A Literature Review

**DOI:** 10.3390/dj13100439

**Published:** 2025-09-24

**Authors:** Adina Nemeș, Diana Voskuil-Galos, Olimpia Bunta

**Affiliations:** 1 Medical Oncology Department, Faculty of Medicine, Iuliu Hatieganu University of Medicine and Pharmacy, 400012 Cluj-Napoca, Romania; adina.nemes@umfcluj.ro (A.N.);; 2 Medical Oncology Department, Prof. Dr. Ioan Chiricuta Oncology Institute, 400015 Cluj-Napoca, Romania; 3 Orthodontics Department, Faculty of Dental Medicine, Iuliu Hatieganu University of Medicine and Pharmacy, 400012 Cluj-Napoca, Romania

**Keywords:** tyrosine kinase inhibitors, renal cell carcinoma, oral mucositis, dysgeusia, xerostomia, osteonecrosis of the jaw, glossitis, burning mouth syndrome, gingival bleeding, kidney cancer

## Abstract

Tyrosine kinase inhibitors (TKIs), either as single agents or in combination with other drugs, have become a gold standard in many oncological pathologies. The identification, analysis, and clinical management of their multiple and various systemic adverse events are a clear requirement and represent a true challenge in daily practice. For this narrative review, registration clinical trials that have led to the approval of certain TKI protocols in the setting of renal cell carcinoma (RCC) were identified via the latest version of the National Comprehensive Cancer Network (NCCN) kidney cancer guidelines. The following keywords were used: Axitinib, Cabozantinib, Lenvatinib, Pazopanib, Sorafenib, Sunitinib, and Tivozanib. RCC therapies have been proven to frequently induce oral symptoms and pathologies such as stomatitis, dysgeusia, xerostomia, osteonecrosis of the jaws, oral dysesthesia, geographic tongue, and dental and periodontal damage. The aim of this review is to emphasize the mechanisms of these common drug-induced buccodental toxicities associated with TKI therapies in RCC and to draft a general clinical management of these adverse events, in order to improve patients’ quality of life and treatment adherence.

## 1. Introduction

**Figure 1:**
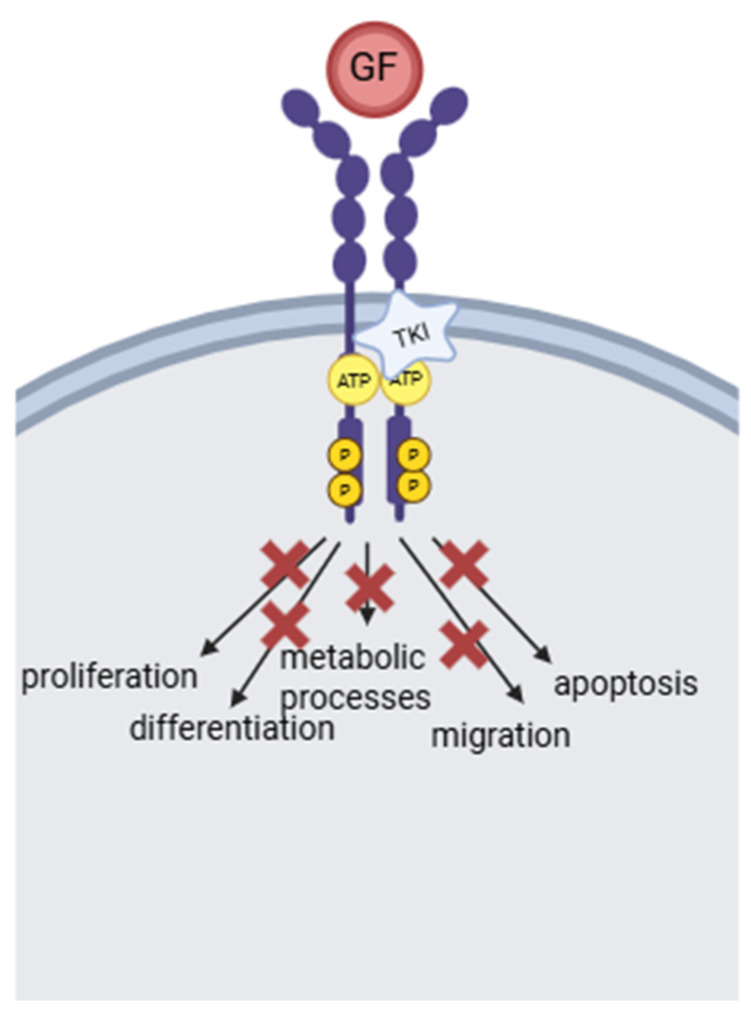
Mechanism of action of tyrosine kinase inhibitors. GF: growth factor; ATP: adenosine triphosphate; TKI: tyrosine kinase inhibitor; and P: phosphorylated protein.

Ongoing developments in novel cancer treatments have led to the approval of tyrosine kinase inhibitors (TKIs) as pharmaceutical agents active against numerous oncological conditions. TKIs represent molecules that block tyrosine kinase enzymes by binding to the catalytic domain of the tyrosine kinase, leading to the inhibition of autophosphorylation and further activation of the enzyme [1,2] (Figure 1). Physiologically, tyrosine kinase enzymes permit proper cell activity, mediating important cellular functions such as proliferation, differentiation, migration, metabolic processes and lastly, apoptosis [3,4]. However, following genetic alterations, tumor cells exhibit aberrant signaling transduction pathways emerging from tyrosine kinases, leading to cell malfunction and, therefore, malignancy [5]. In this context, TKIs prevent abnormal responses of the altered enzyme by disrupting the pathways modulating tumor cell development [1]. Currently, there are more than 70 FDA-approved TKIs for the treatment of solid neoplasms, hematological malignancies, as well as non-malignant inflammatory pathologies [6]. These small molecules have proved to be active against numerous primary targets, with most drugs demonstrating inhibition against multiple protein kinases [6].

Renal cell carcinoma (RCC) accounts for over 4% of new cancer cases diagnosed yearly, and it affects twice as many males as females, with a median age at diagnosis of roughly 65 years [7]. Most of the RCC cases are diagnosed in an early stage [8]. In early stages the disease remains restricted to the kidney and is amenable to local ablative techniques such as surgical intervention (partial or radical nephrectomy) or novel loco-regional techniques such as radiofrequency ablation, cryoablation, or microwave ablation [9]. Approximately 16% of patients are diagnosed in locally advanced stages, when the kidney tumor presents direct spread to regional lymphatic nodules and surrounding tissues [10]. A proportion of patients (up to 13%) will, however, present with metastatic disease from diagnosis [11].

Before the year 2004, patients diagnosed with metastatic RCC (mRCC) would have only benefited from systemic therapy in the form of Interleukin-2 (IL-2) and Interferon-alpha (IFN-α), either as single agents or in combination with chemotherapy [12,13,14]. At the time when IL-2 and TNF-α became the standard systemic therapy for mRCC, the molecules showed superior efficacy to the chemotherapy and hormonal therapy anciently administered as treatment in this setting [14]. Nevertheless, these immunomodulating cytokines would prove minimal response rates of 5–10% [15]. Since then, treatment options for advanced RCC and mRCC have entered a new era, firstly dominated by the discovery of targeted therapies in the form of TKIs and later broadened by the introduction of immune checkpoint inhibitors (ICIs) as active agents against cancerous cells [16]. These novel drugs display improved response rates when administered as single agents, as well as in various combinations between TKIs and immunotherapy or between two ICIs [16,17]. When administering TKIs as treatment for RCC, the primary therapeutic target is the receptor family of vascular endothelial growth factors (VEGFs), consisting of membrane receptor tyrosine kinases 1, 2, and 3 (VEGFR-1, -2, and -3) [18]. TKIs inhibit VEGF–receptor kinases, therefore blocking neoangiogenesis, and may additionally exhibit inhibition towards other signaling pathways that advance tumor development [18]. The main antiangiogenic TKIs recommended as treatment for RCC either as single agents or in combination with other therapeutic classes of drugs are presented in Table 1.

Despite their wide use as treatment for various malignancies, TKI administration is burdened by adverse events affecting multiple organs.

**Figure 2:**
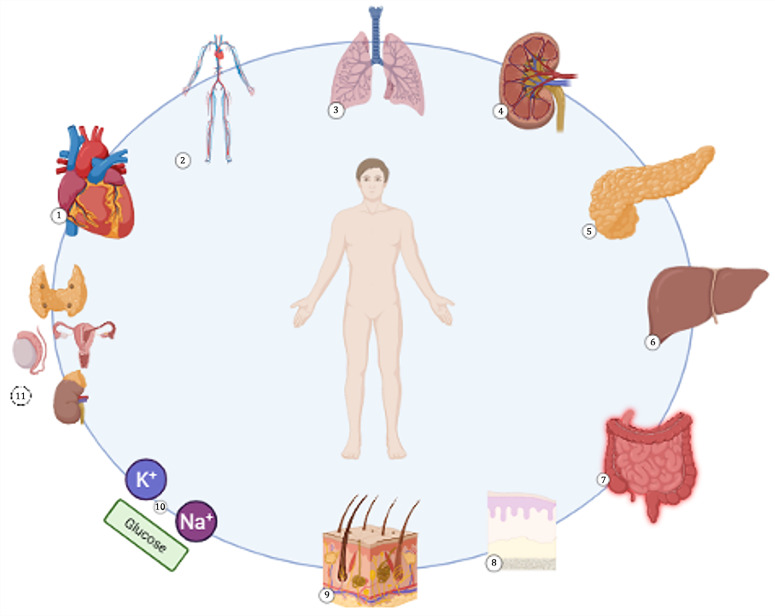
Toxicities induced by tyrosine kinase inhibitors: 1. Cardiac toxicity; 2. Circulatory toxicity; 3. Respiratory toxicity; 4. Kidney toxicity; 5. Pancreatic toxicity; 6. Liver toxicity; 7. Intestinal toxicity; 8. Mucosal toxicity; 9. Skin toxicity; 10. Metabolic toxicity; 11. Endocrine toxicity.

TKI-induced side effects have been reported to involve the cardiocirculatory system, gastrointestinal tract, liver and pancreas, endocrine system, lungs, kidneys, skin and mucosa, and they may induce metabolic abnormalities [1] (Figure 2). Some of the most severe complications observed in clinical practice involve the cardiovascular system and are represented by arterial hypertension, arrhythmias, decreased cardiac output and heart failure, and, most alarmingly, sudden death [29]. Gastrointestinal side effects may also be a source of discomfort and decreased quality of life for patients receiving TKI therapy. Side effects involving the gastrointestinal tract may vary from nausea, vomiting, dyspepsia, anorexia, diarrhea, or constipation to severe forms of colitis warranting immediate intervention [30]. During TKI administration it has been reported that an increase in the levels of hepatic [31] and pancreatic enzymes may occur [32]. TKI-induced endocrine toxicity has been noted more frequently. Patients under treatment with TKI may present altered functions of the thyroid, parathyroid glands, gonads, and, more severely, adrenal insufficiency [33]. Additionally, pulmonary side effects have been noted, mainly presenting as lung fibrosis [34]. Less frequently, TKI therapy may lead to kidney dysfunction as a result of proteinuria, thrombotic microangiopathy, and acute interstitial nephritis [35,36]. Several metabolic imbalances have been associated with TKI use, notably concerning glucose metabolism and electrolyte balance [37,38]. However, one of the most common adverse events during TKI treatment is represented by skin toxicity, frequently presenting as hand-foot syndrome [27].

**Figure 3:**
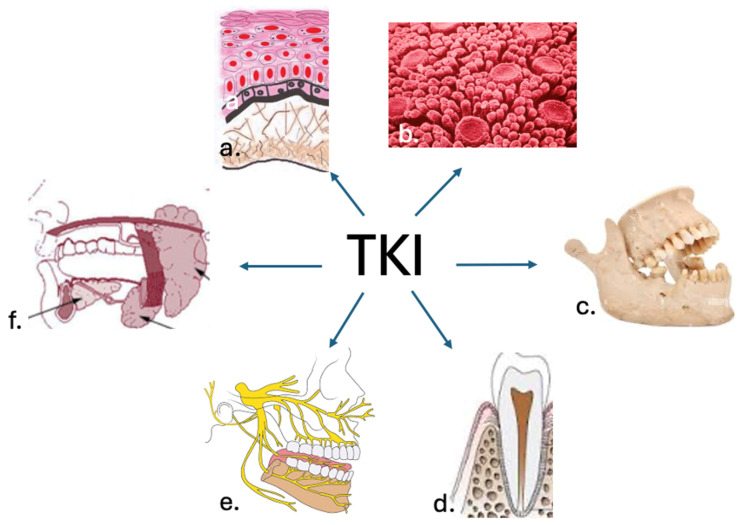
Buccodental structures affected by tyrosine kinase inhibitors: (**a**). oral mucosa; (**b**). tongue; (**c**). jaws; (**d**). teeth and periodontium; (**e**). nerves; (**f**). salivary glands.

Despite being relatively common [39], buccodental toxicities induced by TKI administration require further research. Mucosal injury, also referred to as mucositis, is the most frequently reported oral side effect [40]. However, other oral manifestations have been recorded in clinical practice, consequently influencing patient outcome. Such adverse events also include geographic tongue, oral dysesthesia, dysgeusia, gingival bleeding, xerostomia, osteonecrosis of the jaw, as well as various grades of dental toxicity, particularly pain, instability, or color changes in the teeth [41] (Figure 3).

The precise pathways that lead to these buccodental toxicities have yet to be identified and characterized. VEGFR, PDGFR, and c-KIT are known to play important roles in oral homeostasis [39]. Given the TKI-induced inhibition of these targets, oral complications frequently occur during RCC treatment as a result of mucosal sensitivity and poor healing capacities, impaired cell turnover, and bone deterioration [39,41]. Additionally, taste receptor cells form tissues with high cell regeneration that are commonly affected by cytotoxic drugs, lead to neural outcomes and mucosal signaling distortions [42]. These mechanisms may further explain TKI implications in buccodental clinical symptoms and manifestations.

Some cases of oral toxicity may lack clinical signs at presentation or present with symptoms that can be addressed through lifestyle interventions and minimal conservative approaches. Therefore, clinicians may tend to overlook the significant impact such side effects may have on patients’ quality of life and their motivation to continue specialized therapy. However, oral side effects may lead to an impaired nutritional status, which is linked to a reduced drug efficacy [41]. Additionally, the pain and discomfort associated with oral complications can prompt dose reductions that consequently influence treatment efficiency and may determine poor survival outcomes [41]. Research has also proved that cancer patients show detrimental changes on both physical and psychological levels caused by adverse events in addition to their malignant disease itself [43]. These aspects highlight the importance of better toxicity prevention and screening in cancer patients, as well as the need to better manage each toxicity once it occurs.

The aim of this review is to offer a comprehensive characterization of buccodental toxicities resulting from TKI administration in patients presenting with RCC. In addition, the review provides an analysis of TKI use in the setting of RCC and addresses a general management overview of buccodental adverse events in daily practice with the purpose of raising awareness on the importance of proper recognition and adequate control of these conditions in order to improve patients’ outcomes.

## 2. Materials and Methods

*Search strategy:* In order to achieve the intended purpose of this narrative review, registration clinical trials were extensively studied, including in our research phase 2 and 3 trials that led to the approval of certain TKI protocols in the setting of RCC.

The updated list of registration clinical trials was formulated after consulting the latest version of the National Comprehensive Cancer Network (NCCN) Kidney Cancer guideline (version 1.2026). In order to facilitate the search, known TKIs administered in the setting of RCC were used as key words (Axitinib, Cabozantinib, Lenvatinib, Pazopanib, Sorafenib, Sunitinib, Tivozanib). The information was then selected from the most recent clinical trials addressing the toxicity profile of each TKI in a given administration protocol.

*Data analysis and synthesis:* Once we identified the articles presenting the data obtained during each phase 2 or 3 clinical investigation, particular attention was addressed to the adverse events section of each clinical trial. The content was further analyzed, summarized, and systemized into tables.

*Justification:* We consider the NCCN guidelines as the most current database offering up-to-date knowledge about latest treatments, as well as previous standards of care. Once the NCCN Kidney Cancer guideline was thoroughly studied, the authors were offered a clear research direction that was further elaborated in the narrative section of this review. However, we recognize the limitations of this research due to author flexibility and absence of distinct research protocols.

## 3. Bucco-Dental Toxicities

The randomized clinical trials leading to the approval of the treatment protocols currently employed in RCC management have thoroughly investigated treatment-related adverse events (trAEs). Of the trAEs affecting the buccodental system, stomatitis and dysgeusia have been more extensively covered as side effects occurring with increased prevalence during TKI treatment. Fewer clinical studies have additionally looked into xerostomia and dental involvement as relevant treatment-induced toxicities. Table 2 summarizes buccodental trAEs as observed during late-stage registration clinical trials.

*(a)*  *Mucositis oral/Stomatitis*

Oral mucositis and stomatitis are interchangeable terms used to indicate oral complications of anticancer therapies. “Oral mucositis”, the MeSH term (Medical Subject Headings), and “mucositis oral”, the CTCAE term (Common Terminology Criteria for Adverse Events), both describe inflammation of the oral mucosa as a consequence of chemotherapeutic agents or ionizing radiation. Stomatitis, a more general term, describes any inflammatory condition of the oral tissues [59].

Stomatitis is considered an immune disorder; however, several predisposing factors have been identified. Risk factors include certain foods, stress, genetic predisposition, local tissue injuries, as well as particular classes of pharmacological treatments [60].

In the context of oral aggression, TKI medication may cause oral mucositis as a result of a deficient healing capacity of the mucosa linked to changes in vascular permeability induced by the antiangiogenic activity of multikinase inhibitors [59,61]. Another theory that could explain the development of oral mucositis refers to the decreased capillary structures of the tongue in consequence of VEGF inhibition at this level [62].

The majority of antiangiogenic drugs may induce oral mucositis. Randomized clinical trials studying agents approved as treatment for RCC have noted the prevalence of stomatitis for all TKIs, either as monotherapy or in combination with other classes of drugs (ICIs, mTOR inhibitors). Results show a prevalence ranging from 5% (Sorafenib) [56] to values of >35% (Cabozantinib monotherapy) [47,49] and as high as 47.6% for combination therapy Lenvatinib + Everolimus [24]. However, high-grade toxicity rates are less frequently observed—below 5%, with most TKI administration protocols presenting grade 3 toxicity or higher.

*(b)*  *Dysgeusia*

Dysgeusia is defined as an impaired sense of taste or a distortion of the gustatory perception experienced by patients, who often perceive taste stimuli as bitter, sour, or metallic [63]. While some patients with taste alterations lack an identifiable cause, most of the cases described in the literature can be attributed to one of the following circumstances: age, trauma, surgical injury, neurological conditions, genetic disorders, viral or bacterial infections, and certain medications, particularly those administered as cancer therapies [64]. Depending on the oncological treatment, the prevalence of gustatory alterations in cancer patients ranges from 12% to values as high as 84% [65]. Cancer-induced inflammation, patient nutritional status, and lifestyle, as well as adverse events related to cancer treatment, may contribute to taste modifications in cancer populations [66]. Other possible causes for dysgeusia might be the neurotoxicity linked to certain drugs used to treat cancer, as well as the injury that occurs in tissues with high turnover rates such as those presenting gustatory and olfactory cells [67]. Additionally, other side effects induced by chemotherapy and targeted therapies, particularly stomatitis and xerostomia, could play a role in the development of taste abnormalities [67,68].

Research suggests other causal determinants for TKI-induced dysgeusia, notably the injury sustained by taste and olfactory receptors as a result of continuous chemical drug exposure [68]. VEGFR inhibition showed interference with beta-catenin activity, which plays a key role in the differentiation of receptor cells, leading to an altered renewal of cells responsible for taste perception [69]. Additionally, protein kinase drugs inhibit protein kinase C and Ca2++/calmodulin kinase II involved in olfactory signaling [68]. Damage in VEGFR signaling has also been linked to an accelerated apoptosis and subsequent neurodegeneration that could explain an impaired gustatory stimuli perception [70].

Dysgeusia appears to be an adverse event more frequently studied in new-generation protein kinase inhibitors. Randomized clinical trials investigating Axitinib monotherapy and the combination protocols for Axitinib [44,45,46] and Lenvatinib [24,52,53] noted a prevalence of dysgeusia below 20%. Higher rates were observed for Pazopanib [54] and Cabozantinib [47,48,50], with a value as high as 41% for Cabozantinib monotherapy [47]. Grade 3–4 toxicity, however, occurs in less than 1% of the cases.

*(c)*  *Xerostomia*

Xerostomia defines the perception of dryness of the mouth that results from a reduced secretion of saliva [71]. This condition affects the general population at relatively high rates of more than 20% and increases with age [72]. However, xerostomia sensation is particularly common in patients with chronic illnesses and can reach a prevalence of >70% in patients with advanced malignancies, accounting for one of the five most frequently reported symptoms in this population [73]. Salivary hypofunction in cancer populations is mainly attributed to cancer therapies, notably radiotherapy of the head and neck anatomical regions and chemotherapy drugs that can injure the salivary glands [74]. Additionally, many classes of drugs have been associated with dry mouth sensation, of which we note medications frequently administered to cancer patients for therapeutic purposes, as well as for symptom management: antineoplastics, analgesics (particularly opioids), antidepressants, antibiotics, endocrine drugs, antiemetics, and corticosteroids [75].

Several anti-VEGF targeted treatments for RCC may favor xerostomia [76]. The supposed mechanism leading to symptoms of dry mouth explains how antiangiogenic drugs could decrease the blood flow to the salivary glands and consequently result in reduced salivation [77].

Despite the high frequency of xerostomia among cancer patients, few randomized clinical trials have followed this adverse event. Sunitinib [57], Lenvatinib combination protocols [52,53], and Cabozantinib + Nivolumab [51] were some of the treatments associated with various rates of dry mouth sensation, yet grade 3 or higher toxicity remains an exception.

*(d)*  *Dental, periodontal, and bone-related toxicities*

The World Health Organization defines oral health as the condition of the mouth, teeth, and orofacial structures that allows people to complete vital functions such as eating, breathing, or speaking while embracing important psychosocial elements, such as self-confidence, well-being, one’s ability to socialize and carry on daily tasks without pain, unease, or complexity. There are several oral health assessment tools relying on subjective observations (symptoms expressed by patients), as well as objective findings obtained through visual inspection. There are eight categories to be inspected for a proper oral health assessment: lips, tongue, gums and oral tissue, saliva, natural teeth, dentures, oral cleanliness, and potential dental pain [78]. TKI administration may induce changes in various degrees in all categories of buccodental health. Although less frequent, dental modifications such as color changes in the teeth, mobility of different degrees, cavities, and gingival sensitivity may be noted. These adverse events may further lead to eating difficulties, dietary changes, cavities, gingival inflammation and sensitivity, and in severe cases antibiotic treatment or orofacial surgical interventions may be required in order to preserve function or alleviate symptoms [41].

The exact mechanisms that cause TKI-induced dental, periodontal, and bone changes remain to be determined. A study conducted on rabbit models concluded that Sunitinib negatively impacts the osseointegration of titanium implants inserted in the oral cavity. The research attributed the impaired osteogenesis to a reduced bone angiogenesis, otherwise responsible for bone healing following an implant. We could further hypothesize that VEGF inhibition and decreased angiogenesis could explain other dental toxicities that may occur during targeted therapies with protein kinase inhibitors [79].

TKIs were proven to affect bone metabolism, having the cellular effect of decreasing osteoclastogenesis, altering the metabolism of calcium and phosphate and decreasing bone turnover. Even though inhibition of bone formation and bone resorption, by means of interference in the hematopoietic and mesenchymal stem cells, appears to be a class effect, further research is necessary on the impact of these modifications on the oral cavity structures [80]. The effect of Sunitinib has been studied on Wistar rats in order to assess the tissue repair ability of the bone in tooth extraction sites but only in comparison to zoledronic acid—a bisphosphonate [81]. Despite the acknowledged fact that TKIs alter bone metabolism, few studies have focused on exploring the correlation between cancer therapies and orthodontic treatments [82], especially in the elderly, as pre-prosthetic treatment adjuvants. As orthodontic tooth movement relies, among others, on the bone resorption and apposition phenomena [83], the lack of information and the inability to create evidence-based and standardized orthodontic guidelines and protocols for cancer patients undergoing TKIs represents a major gap in the literature [82].

Dental toxicity has been rarely investigated during clinical trials. However, Axitinib + Avelumab protocol was linked to oropharyngeal pain [45], and Lenvatinib + Everolimus was associated with low-grade oral pain and toothache with a prevalence of 10–12% [52].

*(e)*  *Osteonecrosis of the jaw (ONJ)*

Medication-induced jaw osteonecrosis refers to the bone injury occurring in the maxillofacial region and presents as an exposed area of the mandible or maxilla that does not heal within 8 weeks since diagnosis in patients receiving treatment with bisphosphonates, RANKL inhibitors, m-TOR inhibitors, and antiangiogenic drugs [84]. Additionally, research into the FDA Adverse Event Reporting System identified several other drug classes associated with ONJ, including corticosteroids, taxanes, the ICI nivolumab, the CDK 4/6 inhibitor Palbociclib, and immunomodulatory agents Lenalidomide and Pomalidomide [84]. The risk of developing ONJ varies between medications, and patients undergoing cancer treatment may require administration of multiple agents that favor ONJ development, therefore increasing exposure. Factors that favor ONJ can be classified as local and systemic. Local determinants refer to concomitant oral disorders, anatomical factors, and intraoral surgical interventions that concern the alveolar bone. Systemic factors predisposing patients to ONJ development are advanced age, prolonged exposure to bisphosphonates, concomitant use of corticosteroids, genetic susceptibility, and systemic illnesses (diabetes, dialysis, anemia, hyperthyroidism) [85]. Patients receiving treatment for advanced RCC have a 10% risk of ONJ when receiving concomitant therapy with antiangiogenic TKIs and bisphosphonates, and the risk increases with the duration of the treatment [86]. Patients at risk may experience buccodental clinical presentation of ONJ before developing osteonecrosis, presenting symptoms such as tooth mobility or pain or modifications of the mucosa (swelling, erythema, ulceration, sensibility disorders) [87]. In order to facilitate diagnosis and further treatment, MRONJ follows a classification in 4 stages. Stage 0 refers to patients at risk without clinically or radiologically apparent signs of osteonecrosis, who may experience nonspecific symptoms (pain in the orofacial region); stage 1 defines cases presenting exposure of the dead bone or fistula of the bone, with no other clinical signs or symptoms; in stage 2, patients with necrotic bone experience symptoms characteristic of ONJ and may show signs of infection; lastly, stage 3 includes cases with advanced injury of orofacial structures and severe symptoms [88].

Several mechanisms could explain TKI-induced ONJ. Firstly, TKIs cause direct injury to the oral epithelial cells and compromise the buccal microenvironment. The antiangiogenic activity additionally causes micro-infarctions of the bone and the surrounding soft tissues, leading to impaired bone remodeling and wound healing, while microorganisms of the oral cavity favor cell death of these structures. Lastly, TKI may also interfere with the innate and acquired immune system of the host, promoting bone cell dysfunction and damage [88]. Another theory notes that TKI medication could directly affect bone health through changes in the calcium and phosphate metabolism and reduced osteoclast activity [89]. Additionally, TKIs are thought to interfere with the hematopoietic and mesenchymal cell lineages [89].

Registration clinical trials have not looked into MRONJ as toxicity associated with TKI use; however, there is a significant number of MRONJ cases linked to anti-VEGF medication. A systematic review has noted that 7 out of the 17 patients diagnosed with MRONJ included in the study were treated for RCC with one of the following agents: Axitinib, Pazopanib, Sorafenib, or Sunitinib [89]. Another case series identified 25 cases of ONJ related to TKI administration in RCC. Sunitinib was implicated in the majority of cases (84%), Lenvatinib was linked to 8% of the cases of ONJ, while Cabozantinib and Axitinib each accounted for 1 case (4%) [90]. A different study has successfully outlined case reports of ONJ in patients being treated for RCC with one of the antiangiogenic agents Lenvatinib, Sorafenib, Sunitinib, Axitinib, or Pazopanib [91]. Patients diagnosed with RCC frequently undergo additional bisphosphonate therapy associated with antiangiogenic medication, leading to an increased risk of MRONJ as outlined by an Italian publication [92]. The study identified Sunitinib as the most frequently administered anti-VEGF linked to ONJ development [92].

*(f)*  *Glossitis/Geographic tongue*

Geographic tongue, also referred to as migratory glossitis, is an inflammatory condition that commonly affects the dorsal surface of the tongue [93]. The condition presents itself as erythematous patches on the tongue surface of various sizes and shapes that may be bordered by an irregular white colored margin [93,94]. While the main factors leading to the development of migratory glossitis remain to be determined, the condition has been associated with certain drug use and systemic disorders such as hormonal imbalances, diabetes mellitus, respiratory pathologies, or psoriasis [94,95]. The histological examination of the lesions notes filiform papillae atrophy and desquamated epithelium with infiltrates of neutrophils and lymphocytes [96]. Most cases lack symptoms at presentation; however, some patients may present oral cavity discomfort or pain associated with a burning sensation that consequently impair patients’ quality of life [97].

Even if the pathophysiology of this benign condition has not yet been proven, some authors recognize the important role of the VEGF pathway in the maintenance of buccal mucosal homeostasis. Therefore, the inhibition of VEGF receptors could explain migratory glossitis as a side effect of TKI administration [98].

The occurrence of geographic tongue might be a coincidence in patients with RCC undergoing TKI, but some case reports have described geographic tongue-like changes linked to Axitinib, Sunitinib, and Sorafenib [98,99]. Another publication presented the case of migratory glossitis in a patient treated with Pazopanib for metastatic RCC [100].

*(g)*  *Burning mouth syndrome/Oral dysesthesia*

Patients describing a modification in oral sensitivity recognized as abnormal and disagreeable, experiencing these changes for at least 2 h daily over a course of a minimum of 3 months, present a disorder known as oral dysesthesia or burning mouth syndrome (BMS) [101]. Oral dysesthesia is a cranial neuropathy presenting symptoms that vary from mild tingling of the mouth or a numb sensation at this level, to constant, severe, burning pain of the buccal cavity that leads to decreased function [102]. The clinical examination, however, lacks particular physical and paraclinical elements [103]. With a prevalence <2% in the general population, cases of oral dysesthesia are more frequent among female sex patients and people above the age of 50 years old [104]. Even if an exact etiology has not yet been determined, the interaction between orofacial elements, systemic disorders, and other heterogeneous factors may explain the neurophysiological pathways that lead to BMS development and its psychological implications [105].

Despite the general acknowledgement that psychological factors, such as depression and anxiety, are involved in BMS development and persistence [106,107], several theories attempting to explain the oral symptomatology linked to TKI use have been proposed [40,108]. One hypothesis suggests that VEGF is a constituent of the human saliva in physiological conditions, and, therefore, VEGF inhibition induced by the antiangiogenic action of TKIs could explain the disruption in buccal homeostasis and the occurrence of oral adverse events [108].

A retrospective study concluded that mucosal sensitivity is a frequent side effect presented by patients undergoing treatment with TKIs. Of the antiangiogenic drugs approved for RCC management, the highest incidence of oral dysesthesia was reported for Cabozantinib (34.8%), followed by Sunitinib (23%), Sorafenib (14.5%), and lastly Pazopanib (10.6%) [40].

The precise role that psychological disorders play in the etiology of BMS is still unclear, as more information on their influence as primary cause or aggravating factor is still needed [106,107].

*(h)*  *Gingival bleeding*

Gingival bleeding refers to the bleeding induced by minimal gestures directed towards the interface between the teeth and gingivae, occurring during the basic manipulation of the gingival sulcus and its tissue. Gingival tenderness, redness and swelling, gingival bleeding or blood noticeable in saliva, and halitosis are symptoms of gingival disease [109]. The prevalence of self-reported gingival bleeding described in the literature shows wide variations, from 6 to 78%; however, more studies on the matter report similar values of approximately 50–60% [110]. According to dental specialists, there are multiple causes that induce gingival bleeding: bleeding disorders and systemic diseases such as anemia, liver damage and blood cancers, hormonal changes notable during pregnancy, improper buccodental hygiene and ill-fitted dental appliances, deficiencies of various organic compounds such as vitamin C, vitamin K and vitamin B12, dry mouth and tooth or gum infections, and the use of certain medications. The main drug classes responsible for gingival bleeding are anticoagulants and antiplatelets. Additionally, other drug classes have been linked to gingival bleeding, of which we note those associated with cancer treatment: corticosteroids, antidiarrheals, antiemetics, antineoplastic medication (chemotherapy agents, monoclonal antibodies, and molecule inhibitors), immunosuppressants, endocrine therapies, and bone health medication [111].

Randomized clinical trials and real-world practice have determined that anti-VEGF TKIs could be responsible for gingival bleedings as a result of impaired vascular permeability and a deficient mucosal healing capacity caused by their antiangiogenic effects [112,113]. The biological functions of VEGF include, but are not limited to, the activation of the coagulation cascade, vascular homeostasis through an increase in endothelial cell survival span and vascular integrity and angiogenesis resulting from endothelial cell proliferation and migration, enhanced tube formation and permeability [114]. Therefore, the inhibition of VEGF activity justifies an increased risk of bleeding events. Furthermore, TKI-induced side effects such as stomatitis and xerostomia could render individuals more susceptible to gingival lesions and further bleeding.

A study conducted on 116 patients treated for RCC with either Sunitinib or chemotherapy concluded that gingival bleeding occurred in 62% of patients receiving Sunitinib [41]. Case reports have additionally identified Sunitinib and Pazopanib use to be linked to gingival abnormalities [112,113].

## 4. General Management Overview of Buccodental Toxicities

Drug-induced toxicities carry a significant burden for patients undergoing cancer medical care. Therefore, a correct management of adverse events occurring during treatment is necessary in order to preserve patients’ quality of life and their adherence to therapy. As presented by the state-of-the-art literature assessment, there are only general management mentions on therapeutic possibilities of the buccodental toxicities in patients undergoing TKI therapies for RCC.

Treatment of TKI-induced mucositis is required in order to prevent anorexia and fungal superinfection [115]. Basic measures may include proper buccal routine care, including regular mouth washings and particular attention to dentures [115]. Some cases of mucositis may require additional pharmaceutical treatment in the form of topical coating agents, corticosteroids and anti-inflammatory medication, with or without local anesthetic in order to treat persistent mucositis and alleviate symptoms [42,115]. Severe forms of mucositis complicated with oral candidiasis necessitate antifungal treatment, with topical or systemic administration [116].

Patients presenting oral dysesthesia during cancer treatment pose a challenge in management. Several therapeutic approaches have been described, mostly including antidepressant and anticonvulsant drugs [117]. Other interventions that may prove beneficial are topical administration of local anesthetics, oral administration of alpha-lipoic acid, as well as treatment with various herbal compounds such as aloe vera, capsaicin, or melatonin [117,118]. Research has also looked into laser therapy, transcranial magnetic stimulation, and behavioral therapy as treatment options that could increase the quality of life of patients describing burning mouth syndrome [118]. In addition to dysesthesia, dysgeusia and xerostomia are other toxicities that lead to oral pain and discomfort, therefore warranting treatment. Prevention of dysgeusia may be achieved with a personalized diet and adequate oral hygiene. When such measures prove insufficient, oral administration of zinc supplements in addition to other herbal agents, as well as pharmacological interventions with antidepressants, anesthetics or anxiolytic medication, proton pump inhibitors, or theophylline may improve patient outcome [119]. Mild symptoms associated with dry mouth may be reduced following buccal stimulation (gum, tablets) and application of oral lubricants, while severe cases of xerostomia can be treated with sialogogue drugs [120].

Jaw osteonecrosis is another toxicity that significantly influences the outcome of patients treated with TKIs, with or without bisphosphonate concomitant administration. Patients who present a less aggressive form of ONJ may be treated with conservative methods that focus on proper buccodental hygiene and treatment of dental and periodontal complications [121]. Such cases may also benefit from the use of antibacterial mouth washes and antibiotic therapy [121]. When these methods fail to relieve the pain associated with ONJ, the surgical removal of the necrotic bone may remain the optimal intervention for symptom resolution [87,121,122]. However, the focus should remain on prevention, with careful dental assessment before treatment initiation, as well as during therapy, in order to screen and treat patients at risk of developing ONJ [122].

Other oral adverse events, such as geographic tongue or dental modifications (teeth color changes and mobility, cavities), may remain asymptomatic and in most cases do not require specific treatment [123]. However, some patients may describe gingival bleeding, oral pain, and food aversions caused by tongue and gingival sensitivity to different tastes or temperatures. Such cases could benefit from dietary interventions and in more severe cases, treatment with analgesics, topical anesthetics and corticosteroids, antihistamines, calcineurin inhibitors, or sucralfate [41,124]. Patients experiencing symptoms concerning buccodental health demand specialized dental interventions in order to minimize complications and manage the side effects induced by cancer therapy [41].

Once toxicities occur during TKI therapy, the medical team must evaluate the benefit of the treatment in relation to its side effects. Patients should be asked about drug-adverse events at each presentation, and extensive questionnaires may be implemented in order to obtain an accurate evaluation of treatment tolerance. Dose adjustments based on standardized protocols should be considered in order to preserve patients’ well-being and adherence to treatment. Severe cases that are not managed with conservative and medical interventions should be considered for treatment discontinuation.

As there are no available protocols on the management of buccodental adverse events determined by TKI therapies in RCC patients, prospective clinical trials are necessary in order to elaborate a clear therapeutic approach, in a collaborative manner, between both oncology and dental specialists. Pretreatment examination of the oral cavity, including dental chart, cavities, periodontal, soft tissue, salivary gland, and prosthodontic assessment, as well as improving dental hygiene and dental treatments pre-TKI therapy would be necessary in order to properly address the buccodental adverse events in oncological patients.

## 5. Conclusions

To conclude, the literature highlights the common occurrence of buccodental toxicities in patients undergoing TKI treatment for RCC. However, despite their significant impact on patients’ quality of life, such adverse events tend to be overlooked in daily clinical practice. Additionally, registration clinical trials do not offer insights into all the oral side effects experienced by patients, therefore prompting the need for case report series to document treatment-related manifestations that present difficulty in assessing and classifying. Furthermore, signs and symptoms of such toxicities are less evident, and dose modifications and treatment disruption are seldom required, as the side effects present as low-grade. In this context, further documentation, toxicity recognition, and development of management protocols for these adverse events are of great importance in order to aid clinicians during patient care planning and improve patients’ adherence to treatment and, consequently, their well-being and disease outcome.

## Figures and Tables

**Table 1:** FDA and EMA-approved small-molecule protein kinase inhibitors for the treatment of RCC.

Drug Name	Targets	Drug Associations	Year of Approval by FDA/EMA	Form of Administration	Dosage	Reference
Axitinib	VEGFR-1, VEGFR-2, VEGFR-3, PDGFR, cKIT	Monotherapy	2012/2012	oral	5 mg BID	[19,20]
		Pembrolizumab	2019/2019			
		Avelumab	2019/2019			
Cabozantinib	MET, VEGFR-1, VEGFR-2, VEGFR-3, AXL, RET, ROS1, TYRO3, MER, KIT, TRKB, FLT3, TIE2	Monotherapy	2012/2016	oral	60 mg OD	[21,22]
		Nivolumab	2021/2021		40 mg OD	
Lenvatinib	VEGFR-1, VEGFR-2, VEGFR-3, FGFR-1, FGFR-2, FGFR-3, FGFR-4, PDGFR-α, cKIT, RET	Pembrolizumab	2021/2021	oral	20 mg OD	[23,24]
		Everolimus	2016/2016		18 mg OD	
Pazopanib	VEGFR-1, VEGFR-2, VEGFR-3, PDGFR-α, PDGFR-β, cKIT	-	2009/2010	oral	800 mg OD	[25]
Sorafenib	VEGFR-2, VEGFR-3, PDGFR-β, CRAF, BRAF, V600E BRAF, cKIT, FLT3	-	2005/2006	oral	400 mg BID	[26]
Sunitinib	VEGFR-1, VEGFR-2, VEGFR-3, PDGFR-α, PDGFR-β, cKIT, FLT3, CSF1R, RET	-	2006/2007	oral	50 mg OD	[27]
					4 weeks on/2 weeks off	
Tivozanib	VEGFR-1, VEGFR-2, VEGFR-3, PDGFR-β, cKIT	-	2021/2017	oral	1.34 mg OD	[28]
					3 weeks on/1 week off	

FDA: The Food and Drug Administration; EMA: The European Medicines Agency; BID: twice a day; OD: once a day; VEGFR: vascular endothelial growth factor receptor; PDGFR: platelet-derived growth factor receptor; MET: hepatocyte growth factor receptor; and CSF1R: colony-stimulating factor 1 receptor.

**Table 2:** Buccodental trAEs observed during registration clinical trials for TKI protocols.

Toxicity (CTCAE Toxicity)														
				Stomatitis (Mucositis Oral)		Dysgeusia (Dysgeusia)		Xerostomia (Dry Mouth)		Trial				
Drug Name	Drug Association	Sample Size per Arm	CTCAE Version	Any Grade (%)	Grade 3–4 Toxicity (%)	Any Grade (%)	Grade 3–4 Toxicity (%)	Any Grade (%)	Grade 3–4 Toxicity (%)	Trial Name	Time Frame	Clinical Cutoff	Trial Phase	Ref.
Axitinib	Monotherapy	*n* = 359	3.0	15%	1%	11%	0%	N/A	N/A	AXIS	15 September 2008–23 July 2010	1 November 2011	3	[44]
	Avelumab	*n* = 434	4.03	25.8%	1.8%	13.1%	0%	N/A	N/A	JAVELIN Renal 101	29 March 2016–19 December 2017	20 June 2018	3	[45]
	Pembrolizumab	*n* = 429	4.0	15.6%	0.7%	11.0%	0.2%	N/A	N/A	KEYNOTE-426	24 October 2016–24 January 2018	-	3	[46]
Cabozantinib	Monotherapy	*n* = 79	4.0	35.9%	5.1%	41%	0%	N/A	N/A	CABOSUN	July 2013–April 2015	11 April 2016	2	[47]
		*n* = 331	4.0	22%	2%	24%	0%	N/A	N/A	METEOR	August 2013–November 2014	22 May 2015	3	[48]
		*n* = 44	4.0	37%	2%	N/A	N/A	N/A	N/A	SWOG 1500	April 2016–December 2019	16 October 2020	2	[49]
	Nivolumab	*n* = 320	4.0	16.9%	2.5%	23.8%	0%	N/A	N/A	CheckMate 9ER	September 2017–May 2019	30 March 2020	3	[50]
		*n* = 47	-	28%	0%	N/A	N/A	36%	0%	-	28 August 2018–20 October 2020	20 January 2021	2	[51]
Lenvatinib	Monotherapy	*n* = 52	4.0	23%	2%	N/A	N/A	12%	0%	-	16 March 2012–19 June 2013	10 December 2014	3	[52]
	Pembrolizumab	*n* = 352	4.03	34.7%	1.7%	12.2%	0.3%	N/A	N/A	CLEAR	13 October 2016–24 July 2019	28 August 2020	3	[24]
		*n* = 145	4.03	37%	0%	13%	0%	12%	0%	KEYNOTE-146	21 July 2015–16 October 2019	18 August 2020	1b/2	[53]
	Everolimus	*n* = 355	4.03	47.6%	6.2%	16.6%	0%	N/A	N/A	CLEAR	13 October 2016–24 July 2019	28 August 2020	3	[24]
		*n* = 51	4.0	29%	0%	N/A	N/A	4%	0%	-	16 March 2012–19 June 2013	10 December 2014	2	[52]
Pazopanib	Monotherapy	*n* = 554	3.0	14%	1%	26%	<1%	N/A	N/A	COMPARZ	August 2008–September 2011	May 2012	3	[54]
		*n* = 290	3	9%	<1%	N/A	N/A	N/A	N/A	VEG105192	April 2006–April 2007	15 March 2010	3	[25,55]
Sorafenib	Monotherapy	*n* = 452	-	5%	0%	N/A	N/A	N/A	N/A	TARGET	-	September 2006	3	[56]
		*n* = 355	3.0	12%	<0.5%	8%	0%	N/A	N/A	AXIS	15 September 2008–23 July 2010	1 November 2011	3	[43]
Sunitinib	Monotherapy	*n* = 375	3.0	25%	1%	N/A	N/A	11%	0%	-	August 2004–October 2005	15 September 2005	3	[57]
Tivozanib	Monotherapy	*n* = 173	4.03	18%	2%	N/A	N/A	N/A	N/A	TIVO-3	24 May 2016–14 August 2017	4 October 2018	3	[58]

CTCAE: Common Terminology Criteria for Adverse Events.

## Data Availability

No new data were created or analyzed in this study.

## Abbreviations

The following abbreviations are used in this manuscript:

TKI(s)	Tyrosine kinase inhibitor(s)
(m)RCC	(Metastatic) renal cell carcinoma
FDA	The Food and Drug Administration
IL-2	Interleukin-2
IFN-α	Interferon alpha
ICIs	Immune checkpoint inhibitors
VEGF(R)	Vascular endothelial growth factor (receptor)
PDGFR	Platelet-derived growth factor receptor
NCCN	National Comprehensive Cancer Network
EMA	The European Medicines Agency
trAEs	Treatment-related adverse events
mTOR	Mammalian target of rapamycin
RANKL	Receptor activator of nuclear factor kappa-beta ligand
BMS	Burning mouth syndrome
(MR)ONJ	(Medication-related) osteonecrosis of the jaw
BID	Twice a day
OD	Once a day
MeSH	Medical Subject Headings
CTCAE	Common Terminology Criteria for Adverse Events
